# High expression of acidic chitinase and chitin digestibility in the stomach of common marmoset (*Callithrix jacchus*), an insectivorous nonhuman primate

**DOI:** 10.1038/s41598-018-36477-y

**Published:** 2019-01-17

**Authors:** Eri Tabata, Akinori Kashimura, Maiko Uehara, Satoshi Wakita, Masayoshi Sakaguchi, Yasusato Sugahara, Terumi Yurimoto, Erika Sasaki, Vaclav Matoska, Peter O. Bauer, Fumitaka Oyama

**Affiliations:** 1 Department of Chemistry and Life Science, Kogakuin University, Hachioji, Tokyo, 192-0015 Japan; 2 Research Fellow of Japan Society for the Promotion of Science (DC1), Koujimachi, Chiyoda-ku, Tokyo, 102-0083 Japan; 3 Central Institute for Experimental Animals, Tonomachi, Kawasaki, Kanagawa 210-0821 Japan; 4 Laboratory of Molecular Diagnostics, Department of Clinical Biochemistry, Hematology and Immunology, Homolka Hospital, Roentgenova 37/2, Prague, 150 00 Czech Republic; 5 Bioinova Ltd., Videnska 1083, Prague, 142 20 Czech Republic

## Abstract

Chitin is a polymer of *N*-acetyl-D-glucosamine (GlcNAc) and a main constituent of insects’ exoskeleton. Insects are rich in protein with high energy conversion efficiency. Recently, we have reported that acidic chitinases (Chia) act as digestive enzymes in mouse, pig and chicken (omnivorous) but not in dog (carnivorous) and bovine (herbivorous), indicating that feeding behavior affects Chia expression levels, and determines chitin digestibility in the particular animals. Common marmoset (*Callithrix jacchus*) belongs to New World monkey family and provides a potential bridge between mouse models and human diseases. Common marmoset is an insectivorous nonhuman primate with unknown expression levels and enzymatic functions of the Chia homologue, CHIA. Here, we report that common marmoset highly expresses pepsin-, trypsin- and chymotrypsin-resistant CHIA in the stomach. We show that CHIA is most active at pH 2.0 and degrades chitin and mealworm shells into GlcNAc dimers under gastrointestinal conditions. Although common marmoset and crab-eating monkey (Old World monkey) have two CHIA genes in their genomes, they primarily express one gene in the stomach. Thus, this study is the first to investigate expression levels and enzymatic functions of CHIA in a New World primate, contributing to the understanding of dietary adaptation and digestion in this taxon.

## Introduction

Chitin is a polymer of β-1, 4-linked *N*-acetyl-D-glucosamine (GlcNAc). It is main constituent of chitin-containing organisms such as crustaceans, insects and fungi^1–3^ and is the second most abundant polysaccharide in the nature. Although humans and mice do not synthesize chitin, they produce two active chitinases^2,4–6^. Chitotriosidase (CHIT1) is markedly increased in Gaucher disease patients^7–9^. Acidic chitinase (hereafter referred to as “CHIA” in primates or “Chia” in other animals; also reported as acidic mammalian chitinase, AMCase) gained its name due to its acidic isoelectric point^10^. CHIT1 and CHIA have been regarded as having protective role against chitin-containing pathogens^2,6^.

CHIA has attracted considerable attention because CHIA levels are markedly altered in various diseases such as asthma, allergic inflammation, gastric cancer, ocular allergy and dry eye syndrome^11–17^. Polymorphisms and certain haplotypes of Chia have been shown to be associated with bronchial asthma in humans^18–20^. Recently, it has been shown that Chia is required for airway chitinase activity in mouse^21,22^. In addition, Chia functions as a critical initiator of protective type 2 responses to intestinal nematodes in mouse^23^.

Since chitin has long been considered as a dietary fiber that is not processed in the digestive system, it has been included occasionally in animal feeds^24^. Recently, we have shown that Chia proteins are abundantly expressed in the stomach of mouse, pig and chicken (omnivorous animals). Chia is resistant to digestion by pepsin at pH 2.0 as well as trypsin and chymotrypsin at pH 7.6, while its chitinolytic ability is preserved under either gastrointestinal tract (GIT) condition. Chia degrades colloidal and crystalline chitin and produced (GlcNAc)_2_ fragments, which are likely a good source of carbon, nitrogen and energy for the animals^25–27^. In contrast, herbivorous and carnivorous animals such as bovine and dog have very low capability to digest chitin when compared to omnivorous animals^28^. Furthermore, some herbivorous animals such as rabbit and guinea pig do not contain functional Chia genes^28^. Recently, it has been reported that nonhuman primates, including common marmoset, retain several CHIA genes and that species with higher insect consumption have up to five CHIA genes in their genome as revealed by whole genome sequencing^29^. Other recent expansive genetic study also suggests that CHIA expression in placental mammals, including primates, are related to feeding behavior^30^. Thus CHIA genes may have been subjected to selection based on diet^28–30^.

Common marmoset (*Callithrix jacchus*), which belongs to New World monkey family, has been attracting a lot of attention in biomedical research because of its biological similarities to human, comparative ease in handling due to its small size and high reproductive efficiency^31–36^. Common marmoset provides a potential bridge between mouse models and human disorders^31–36^. They inhabit humid Atlantic forest of north-eastern Brazil and are consuming fruits, flowers, plant exudates (gums, saps, latex) and insects^34^.

Since insects are ubiquitous organisms and are rich in protein with high energy conversion efficiency^37,38^, they are an important component of the nonhuman primate diets. However, it remains to be determined whether and how the CHIA genes are transcribed, and whether CHIA proteins can function as digestive enzymes in common marmoset.

Here, we report that common marmoset highly expresses CHIA in the stomach, which can digest insect chitin. Also, we show that one of the CHIA gene encoded in the genome is primarily expressed in the stomach. Our results provide important insights to clarifying nutritional values and physiological effects of insects as well as the relationship between feeding behavior and molecular evolution of CHIA in nonhuman primates.

## Results

### CHIA is expressed in a tissue-specific manner in common marmoset stomach

We investigated the expression patterns of CHIA mRNA in ten normal common marmoset tissues (brain, salivary, lung, heart, stomach, intestine, colon, liver, kidney and spleen). We constructed a marmoset standard DNA containing cDNA fragments of CHIA, CHIT1, glyceraldehyde-3-phosphate dehydrogenase (GAPDH), pepsinogen A (Pep A) and H^+^/K^+^-ATPase in a one-to-one ratio (Supplementary Fig. S1) and performed gene expression analysis using a quantitative reverse transcriptase-coupled PCR (qPCR) assay as described in the Methods. This qPCR system enabled us to quantify and compare the gene expression levels of the chitinases and the reference genes on the same scale^39,40^.

To evaluate the quality of RNA and chitinase levels, the housekeeping gene GAPDH was set as the reference gene^41,42^ (Supplementary Table S1). The detected levels of GAPDH in all tested tissues indicated cDNA quality suitable for further analysis of the gene expression pattern.

**Figure 1:**
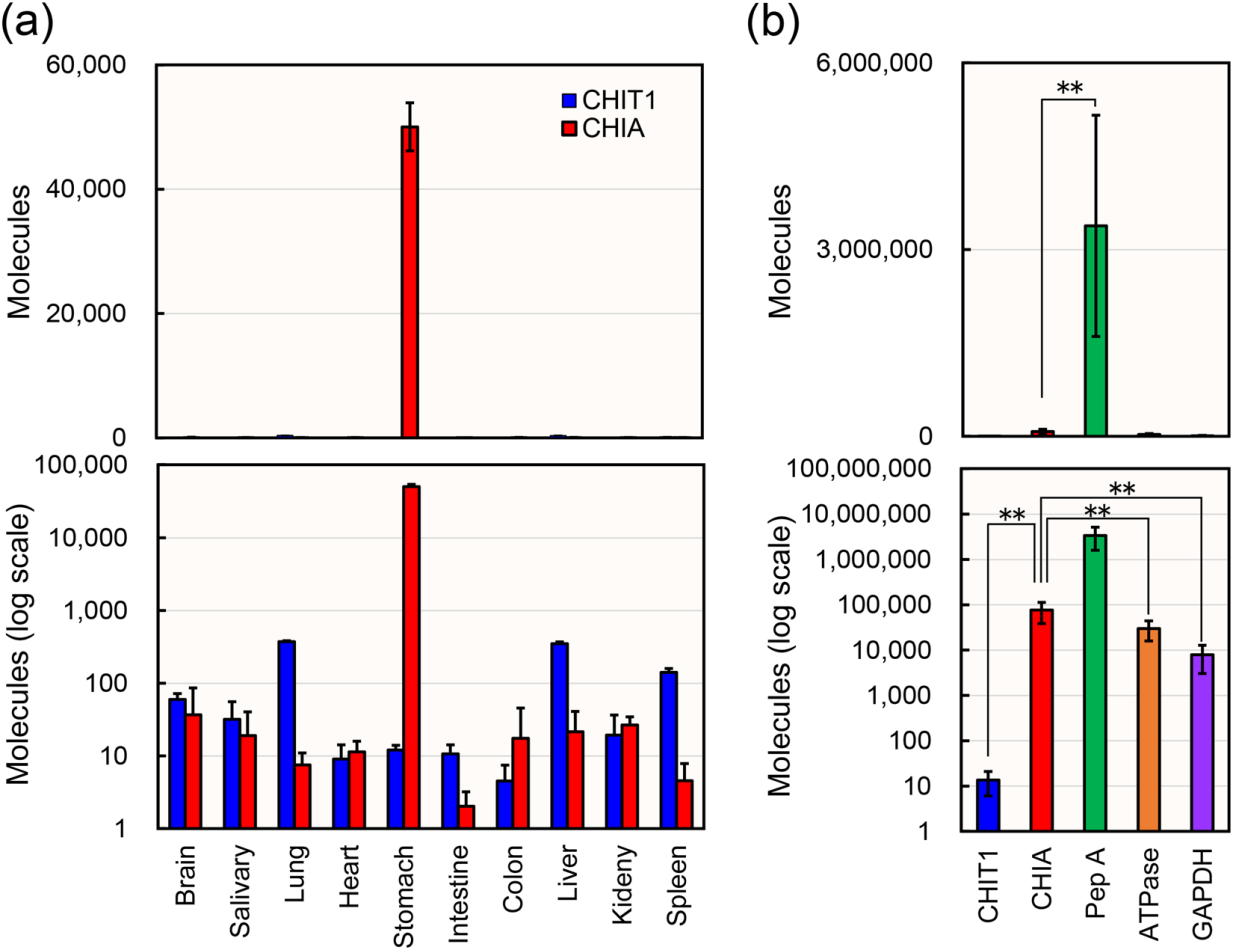
Expression of CHIT1 and CHIA mRNAs in common marmoset tissues. (**a**) Quantification of CHIT1 and CHIA mRNAs in different common marmoset tissues. The marmoset tissues were dissected from a 7-year-old female marmoset. Chitinases were quantified by qPCR using the standard DNA. (**b**) Evaluation of CHIA mRNA levels in the six marmoset stomach tissues using standard DNA containing five genes fragments: CHIA, CHIT1, GAPDH, pepsinogen A (Pep A) and H^+^/K^+^-ATPase. The stomach tissues were dissected from a seven-year-old female and eight or ten-year-old male marmosets. Y axis represents molecules per 10 ng of total RNA. All mRNA copy numbers were calculated based on the same standard DNA dilutions. Upper panel, actual value; lower panel, logarithmic scale. Data are represented as mean ± SD from a single experiment conducted in triplicate. **p < *0*.*01*. We determined P-values using Student’s t-test.

The relatively high levels of CHIT1 mRNA were detected in the lung, liver and spleen (Fig. 1a, lower panel) although they were lower than those of GAPDH (Supplementary Table S1). In contrast, CHIA is highly expressed in stomach, exceeding the levels of CHIT1 and GAPDH (Fig. 1a, upper panel; Supplementary Table S1). In other tissues, the levels were similar or lower than those of CHIT1 (Fig. 1a, lower panel). Thus, clear tissue-specific pattern was observed in CHIA mRNA expression.

To clarify more precisely the expression pattern in the common marmoset stomach (Fig. 1a) with high CHIA level, we analyzed stomach cDNAs prepared from six animals using our qPCR system. The mRNAs of major gastric proteins (pepsinogen A, H^+^/K^+^-ATPase) as well as GAPDH were used as reference genes. Pepsinogen A is a precursor of pepsin A, a major digestive enzyme^43^, whereas H^+^/K^+^-ATPase functions to acidify the stomach^44^. CHIA mRNA level was found to be significantly higher than those of CHIT1, H^+^/K^+^-ATPase and GAPDH, while 44 times lower than that of pepsinogen A in all animals (Fig. 1b; Supplementary Table S1). These results indicate that CHIA mRNA is highly expressed in the common marmoset stomach tissues.

### CHIA is resistant to endogenous pepsin treatment

**Figure 2:**
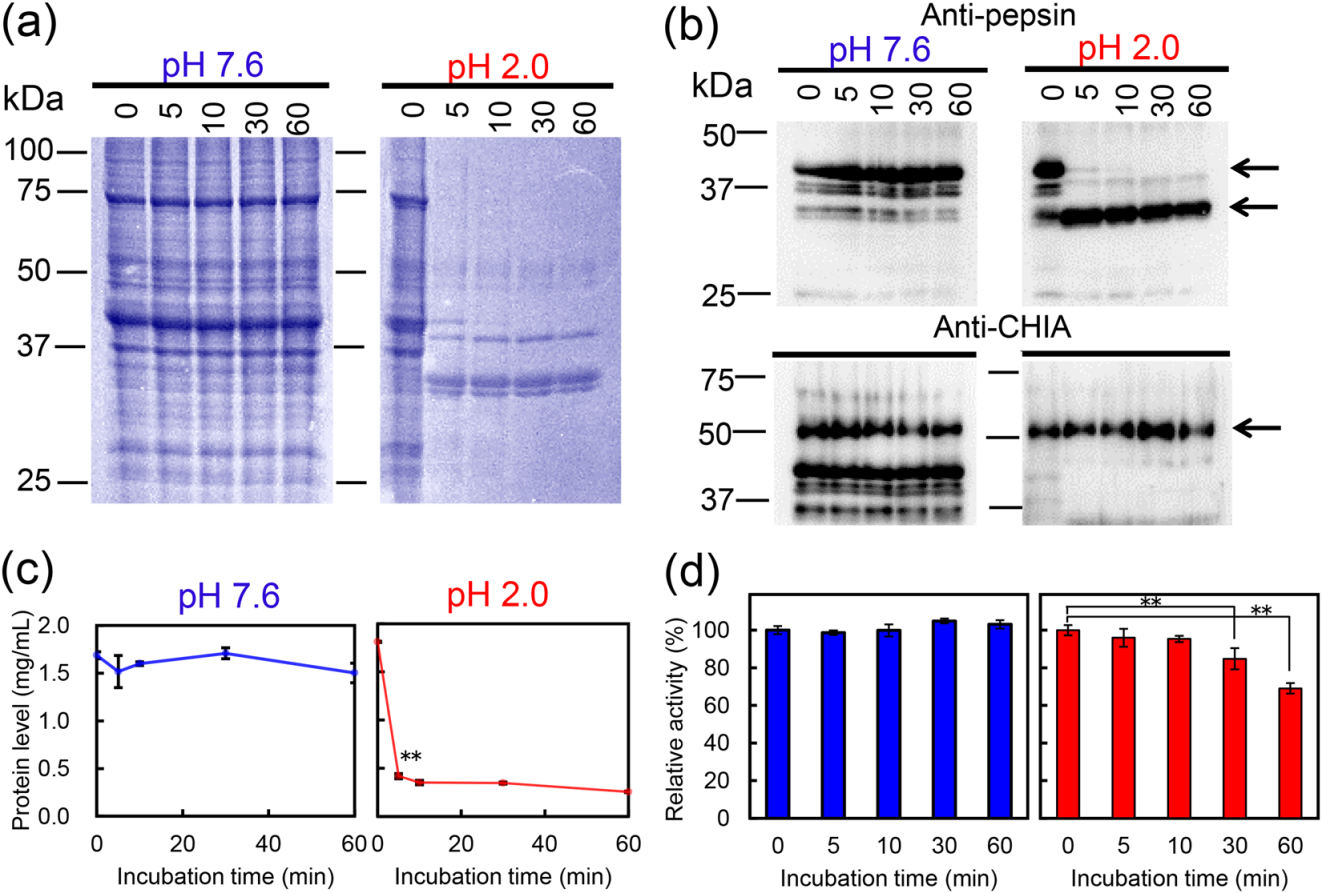
CHIA is not degraded by endogenous pepsin. Soluble proteins obtained from common marmoset stomach were incubated at 37 °C for 0, 5, 10, 30 and 60 min at pH 7.6 or 2.0. (**a**) Total protein analysis by Coomassie Brilliant Blue (CBB) staining, (**b**) Western blot, (**c**) total protein levels quantification and (**d**) chitinolytic activity assay measured at pH 2.0 as described in the Methods. Values in (**c**,**d**) represent mean ± SD from a single experiment conducted in triplicate. **p < 0.01. P-values were determined using Student’s t-test. The images of (**a**,**b**) were cropped from original full-length gel images with same exposure time shown in Supplementary Fig. S4.

Next, we examined whether CHIA is resistant to treatment by endogenous pepsin in artificially created common marmoset stomach condition at pH 2.0 and 37 °C. We prepared soluble protein fraction from the marmoset gastric tissue without protease inhibitor and incubated the fraction at pH 7.6 or pH 2.0 for 60 min. The treated fraction was analyzed by SDS-polyacrylamide gel electrophoresis (PAGE), followed by Coomassie Brilliant Blue (CBB) staining. We found that no changes in the band pattern and intensities were noticed within 60 min incubation at pH 7.6 (Fig. 2a), while time-dependent decrease of total soluble protein was observed after as early as 5 min of incubation at pH 2.0 with several bands remaining unmodified even after 60 min (Fig. 2a). We next carried out Western blot using anti-pepsin antibody and noticed a shift of the respective bands within 5 min of incubation at pH 2.0, indicating the pepsinogen-to-pepsin conversion (Fig. 2b, arrows). The pepsin level remained virtually unchanged at pH 2.0 during the 60 min incubation (Supplementary Fig. S2).

We next evaluated the marmoset CHIA using anti-pig Chia antibody (Supplementary Fig. S3) by Western blotting. The antibody recognized a band at 52 kDa (Fig. 2b). Although the amount of the total protein rapidly decreased by acid denaturation and pepsin digestion at pH 2.0 (Fig. 2c), the 52 kDa band was not significantly affected during the 60 min incubation (Figs 2b and S2). Then, we examined the chitinolytic activity using 4-nitrophenyl *N*,*N′*-diacetyl-β-D-chitobioside (4-NP-chitobioside) and found that the reduction of chitinase activity was slow (Fig. 2d). These results indicated that marmoset CHIA (52 kDa protein), is stable in the presence of pepsin at pH 2.0 and maintains its chitinolytic activity.

### Characterization of enzymatic activity of common marmoset CHIA

**Figure 3:**
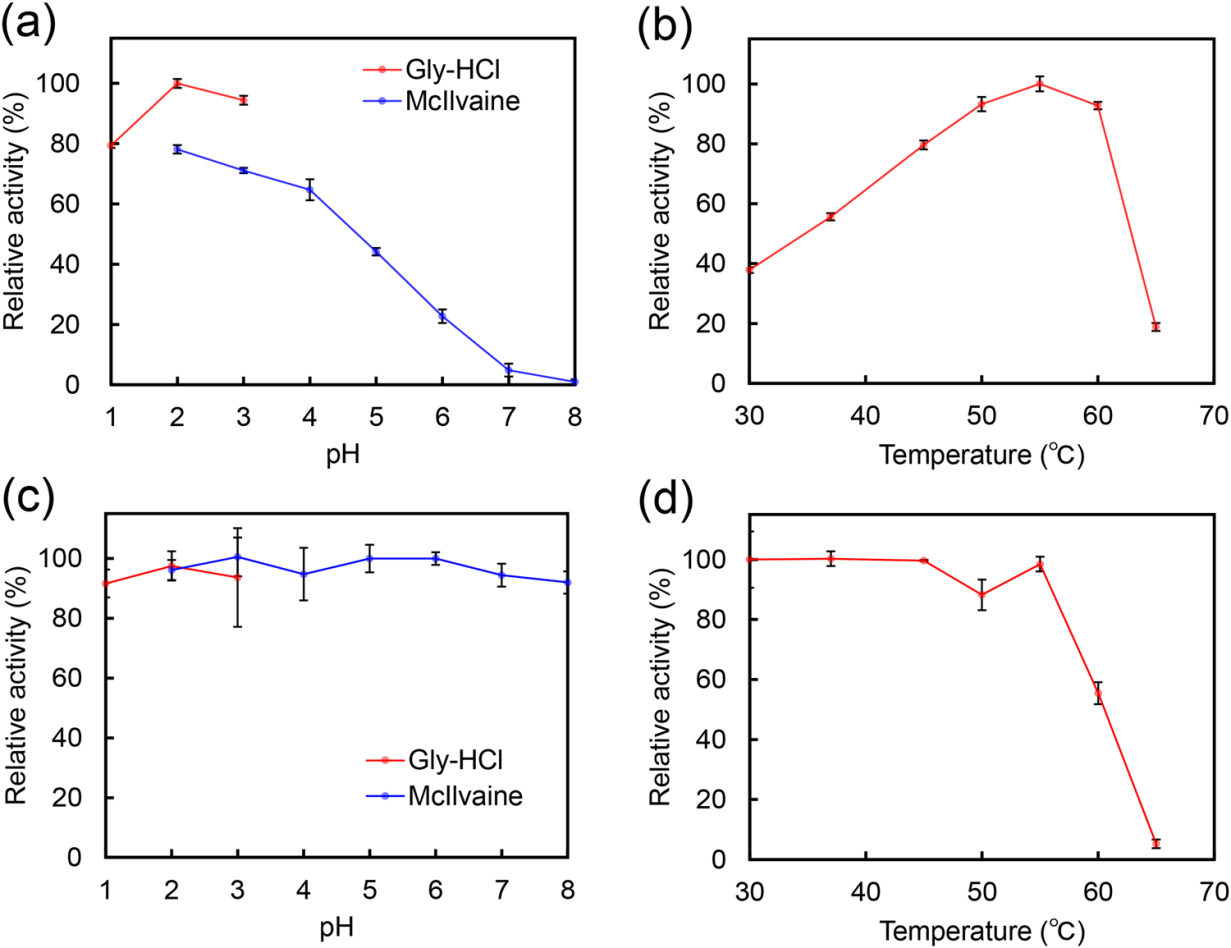
Characteristic of common marmoset CHIA. Chitinolytic activity was measured using 4-NP-(GlcNAc)_2_ in the stomach extract. (**a**) Optimal pH: the chitinase activity was investigated in Gly-HCl buffer (pH 1.0 to pH 3.0) or McIlvaine’s buffer (pH 2.0 to pH 8.0) at 37 °C for 30 min. (**b**) Optimal temperature: chitinase activity was assayed between 30 °C and 65 °C in Gly-HCl buffer (pH 2.0). (**c**) pH stability: samples were incubated for 1 hour on ice in 0.1 M Gly-HCl buffer (pH 1.0 to pH 3.0), McIlvaine’s buffer (pH 2.0 to pH 8.0). After the pre-incubation at the indicated pH, the residual activity was analyzed at pH 2.0 in 0.1 M Gly-HCl buffer, as described above. (**d**) Thermal stabilities: samples were incubated at pH 2.0 in Gly-HCl buffer for 15 min between 30 °C and 65 °C. After cooling on ice, the residual activity was measured at pH 2.0 in 0.1 M Gly-HCl buffer, as described above. Values represent mean ± SD.

Using 4-NP-chitobioside as the substrate, we determined the pH optima by measuring enzyme activity at different pH in 0.1 M Gly-HCl (pH 1.0–3.0) or McIlvaine’s (pH 2.0–8.0) buffers for 30 min at 37 °C. The highest activity was detected at pH 2.0 and it was decreasing with increasing pH (up to pH 8.0) (Fig. 3a).

We next determined the influence of temperature on enzymatic activity at pH 2.0 at 30 to 65 °C using the same substrate for 30 min in 0.1 M Gly-HCl buffer. As shown in Fig. 3b, the enzymatic activity increased with rising temperature and reached the maximum at 55 °C, then sharply ceased.

The pH stability of the marmoset CHIA was determined using the same substrate. We pre-incubated the enzyme on ice for 60 min at various pH in Gly-HCl or McIlvaine’s buffers. The residual activity was then measured at 37 °C and pH 2.0. The marmoset CHIA had a remarkable acid stability since the pre-incubation at pH 2.0 did not lead to loss of the chitinase activity (Fig. 3c).

Finally, we assessed the thermal stability of the marmoset CHIA with pre-incubation of the samples at pH 2.0 for 15 min at 30 to 65 °C. The residual activity was measured using the chromogenic substrate at 37 °C and pH 2.0. The enzyme appeared heat-stable to up to 55 °C at pH 2.0 (Fig. 3d) while its activity decreased at temperatures above 55 °C.

### Common marmoset CHIA is resistant to intestinal proteases

**Figure 4:**
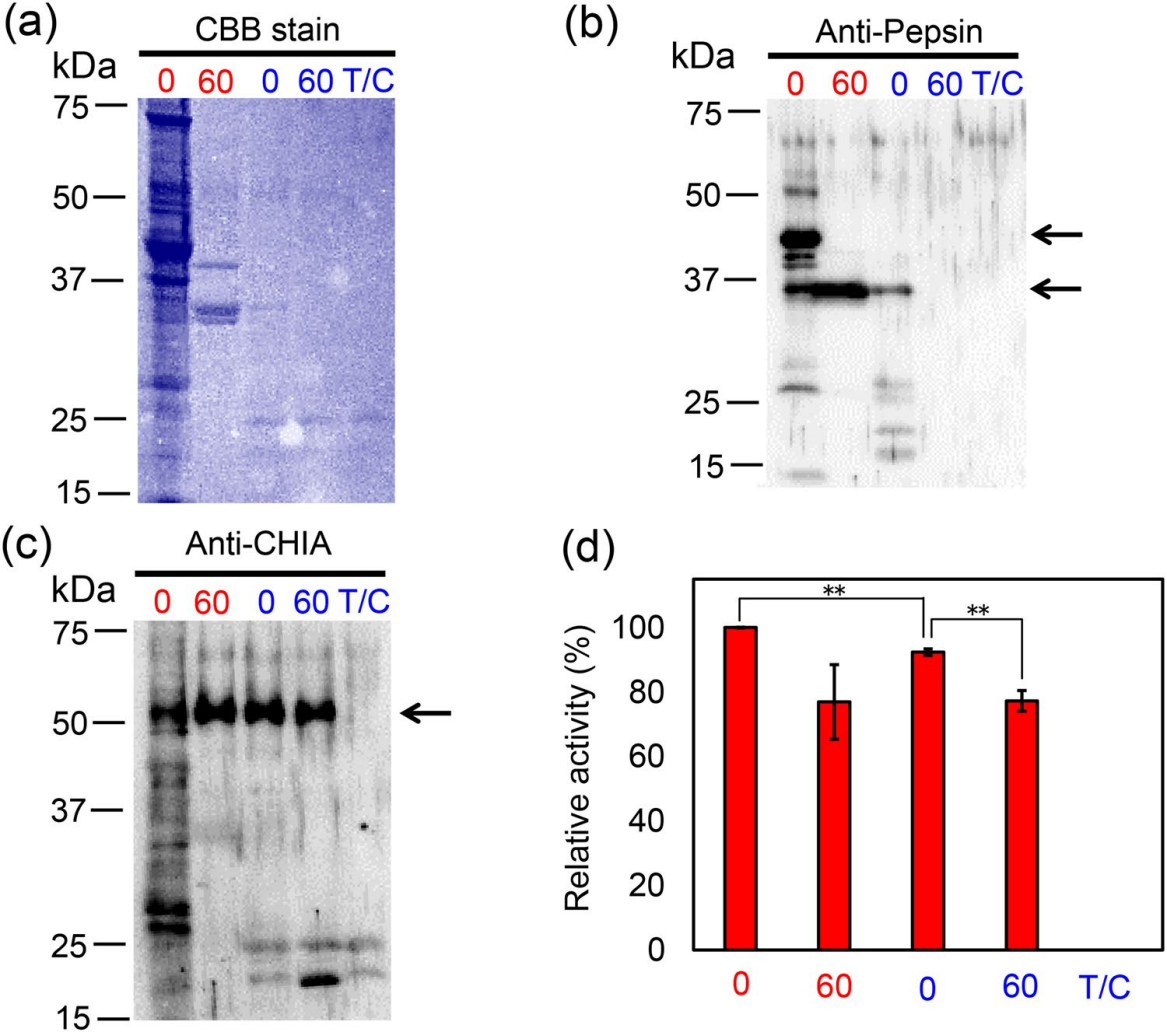
CHIA is resistant to digestion by trypsin and chymotrypsin. Soluble proteins fraction from common marmoset stomach was incubated under stomach-like condition, followed by incubation in intestine-like environment containing trypsin and chymotrypsin at equal concentrations with the soluble stomach proteins. The samples were analyzed by (**a**) CBB staining, (**b**) Western blot using anti-pepsin antibody, (**c**) Western blot using anti-pig Chia antibody and (**d**) chitinolytic activity measurement. All incubations were performed at 37 °C. T/C, trypsin/chymotrypsin only; numbers, incubation time of extract and trypsin/chymotrypsin in minutes under stomach-like (red) or intestine-like (blue) conditions. The images of (**a**–**c**) were cropped from original full-length gel images with same exposure time shown in Supplementary Fig. S6. Values in (**d**) represent mean ± SD, experiments were conducted in triplicate. **p < 0.01 P-values were determined using Student’s t-test.

We next investigated the stability of pepsin and CHIA in the artificial intestinal environment containing trypsin and chymotrypsin. The soluble protein fraction from the stomach was incubated first at pH 2.0 and 37 °C for 1 hour, followed by incubation with equal amount of trypsin/chymotrypsin (0.5 µg) at pH 7.6 for 1 hour. In agreement to the previous experiment (Fig. 2), the total protein drastically decreased during the incubation under at pH 2.0. Subsequent incubation in the presence of trypsin and chymotrypsin under intestine condition at pH 7.6 resulted in its further degradation (Fig. 4a). Pepsin was mostly digested, whereas CHIA levels were not affected (Figs 4b,c and S5). Moreover, the chitinolytic activity also slightly decreased, but had been maintained throughout whole incubation at this condition (Fig. 4d). These data indicate that CHIA is also stable under the intestine conditions.

### Common marmoset CHIA digests chitin substrates mimicking gastrointestinal environment

**Figure 5:**
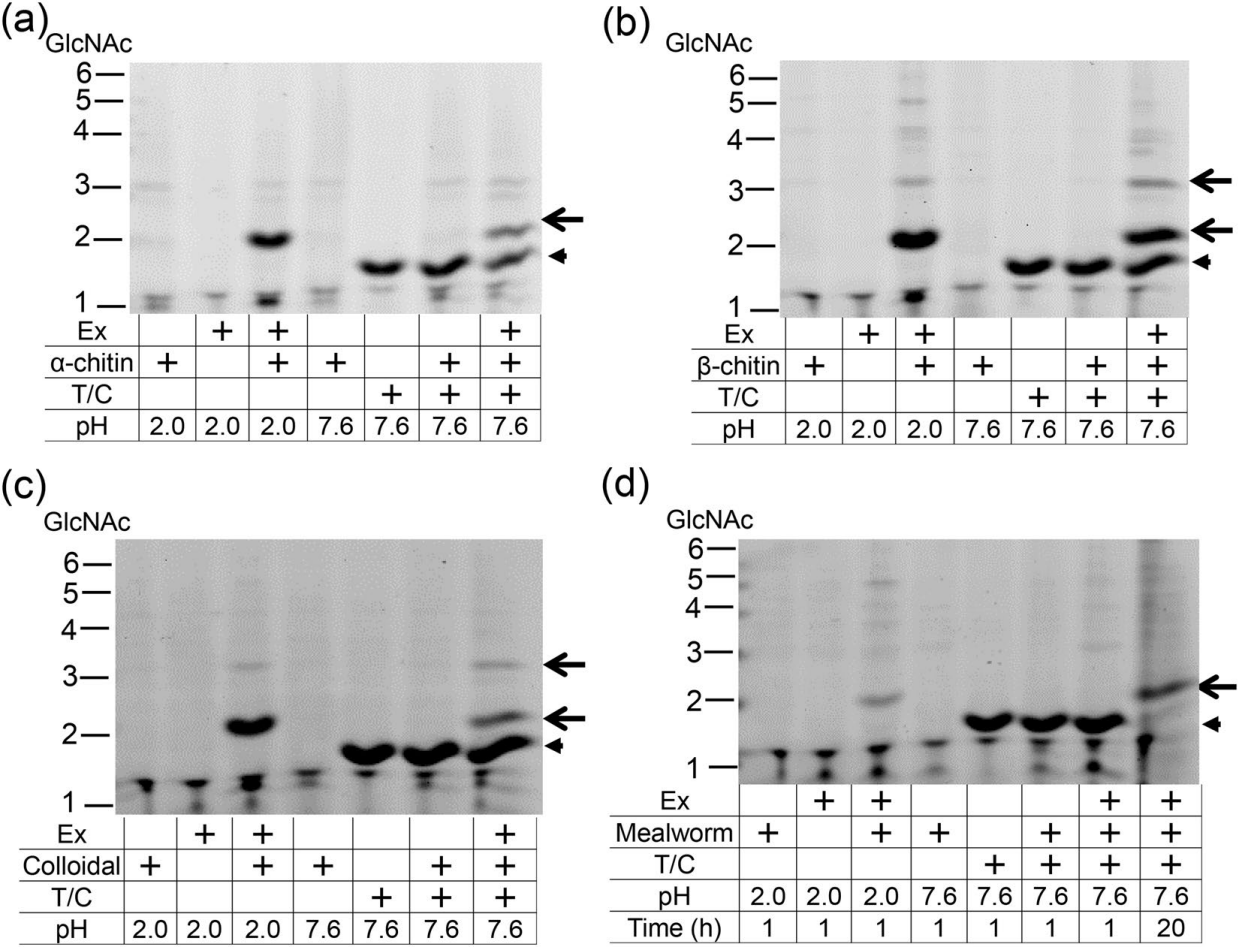
Chitin substrates and mealworm shells are degraded by CHIA under conditions mimicking GIT. Degradation products generated by incubation of (**a**) α or (**b**) β chitin and (**c**) colloidal chitin with soluble protein from common marmoset stomach extract at pH 2.0 or at pH 7.6 supplemented with trypsin and chymotrypsin were analyzed by FACE. (**d**) Mealworm larvae shells is also incubated with soluble protein under mimicking GIT conditions described before. Chitin degradation products are pointed by arrows, and the artifact bands are also indicated by arrowheads. The specific bands between GlcNAc and (GlcNAc)_2_ (shown by arrows) were observed in the samples at pH 7.6. Since the specific bands (shown by arrowheads) are generated after the incubation with trypsin/chymotrypsin (T/C) in the absence of enzyme and substrates, those bands may be artifacts derived from trypsin/chymotrypsin. The images of (**a**–**d**) were cropped from original full-length gel images with same exposure time shown in Supplementary Fig. S7.

Next, we tested whether polymeric chitin substrates can be degraded by CHIA in the gastrointestinal environment. We incubated α, β or colloidal chitin with stomach extract at pH 2.0 or at pH 7.6 with trypsin and chymotrypsin (0.5 µg) (Fig. 5a–c). Also, mealworm shells (*Tenebrio molitor*) were used as chitin-containing organism substrates (Fig. 5d). We analyzed the degraded products by the improved method of fluorophore-assisted carbohydrate electrophoresis (FACE)^45,46^. We found that endogenous CHIA activity in the stomach extract degraded all chitin substrates and produced primarily (GlcNAc)_2_ fragments under both conditions after 1- or 18-hours incubation. Thus, CHIA degrades polymeric chitin substrates under GIT-like conditions.

### Analysis of CHIA genes in common marmoset and crab-eating monkey

**Figure 6:**
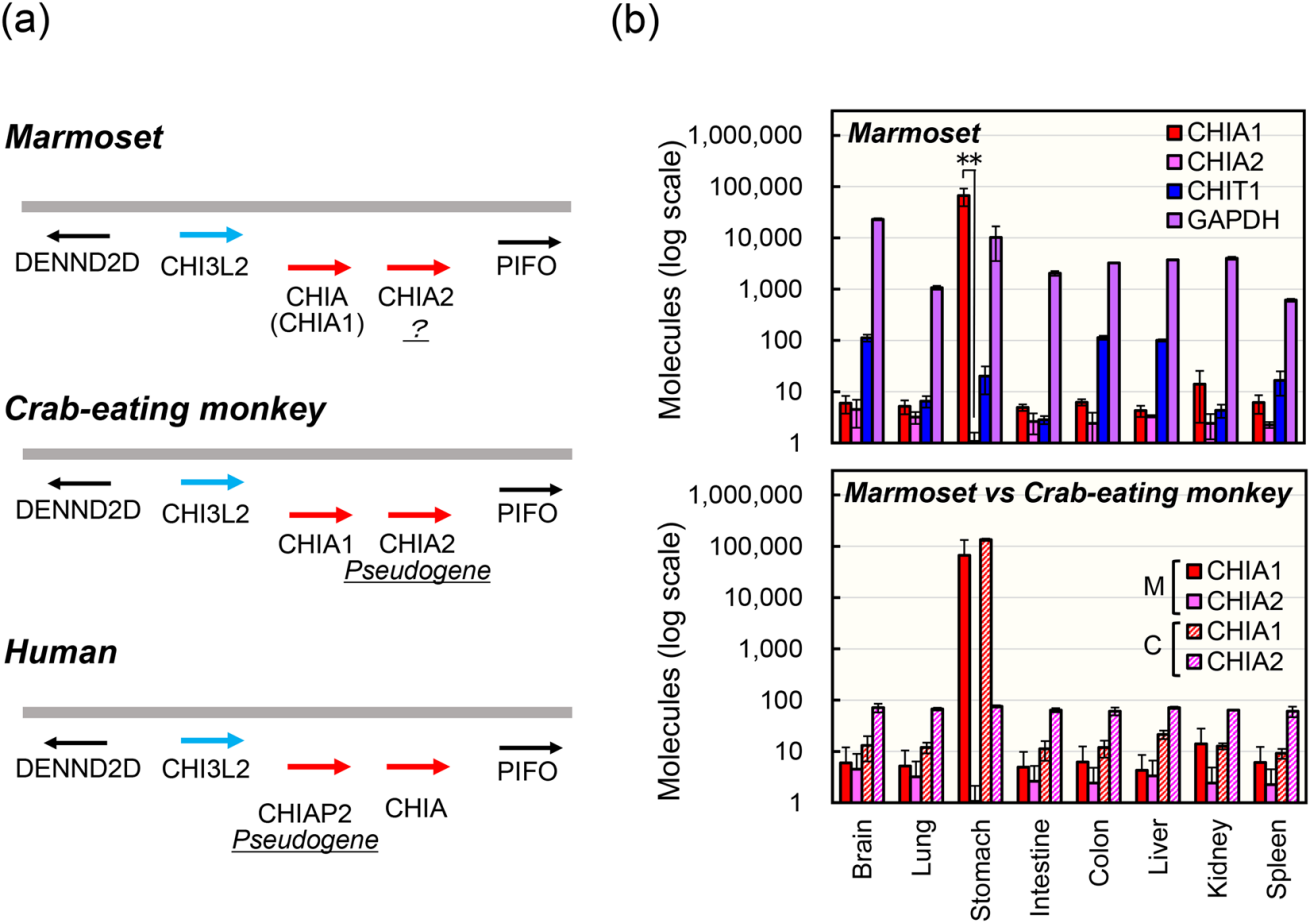
CHIA (CHIA1) is a major transcript in the stomachs of common marmoset and crab-eating monkey. (**a**) Schematic representation of the CHIA genes (red) as well as neighboring marker genes (black or blue). CHIA-like regions can be found between DENND2D and PIFO (black). Common marmoset and crab-eating monkey have CHI3L2 (known as chitinase-like protein, blue), CHIA (CHIA1) and CHIA2 (red) in the region. In human, CHIAP2 and CHIA are in the regions. (**b**) Evaluation of CHIA (CHIA1) and CHIA2 mRNA levels in the marmoset (upper panel) and comparison of those levels between the marmoset and crab-eating monkey (lower panel) tissues using standard DNA containing seven genes fragments: marmoset CHIA (CHIA1), CHIA2, CHIT1 and GAPDH and crab-eating monkey CHIA1, CHIA2 and GAPDH. M, marmoset; C, crab-eating monkey (lower panel). All values are expressed as molecules per 10 ng of total RNA. The panels show the logarithm of the values. Each experiment was performed in triplicate. **p < *0*.*01*. P-values were determined using Student’s t-test.

According to the recent report^29^, primates have several CHIA genes in their genomes. Common marmoset has two CHIA genes, CHIA [here we call CHIA (CHIA1); GenBank Accession Number, XM_017975081.1] and CHIA2 (XM_009001910.1) (Fig. 6a), similarly to crab-eating monkey (Old World monkey) that also has CHIA (CHIA1) (NM_001284548.1) and CHIA2 (XM_015431295.1). In previous reports, these genes were called mCHIA (macaque CHIA) and hCHIA (human CHIA), respectively^29,47^. In human genome, CHIAP2 (NR_003928.2; pseudogene) and CHIA (NM_201653.3) are related to CHIA (CHIA1) and CHIA2, respectively (Fig. 6a). Although CHIA2 has been reported as pseudogene in crab-eating monkey^47^, its expression is unknown in common marmoset.

For analysis of the CHIA2 mRNA in common marmoset and its comparison with the crab-eating monkey as control, we performed qPCR using standard DNA (Supplementary Fig. S8) for internal control in eight major tissues (brain, lung, stomach, intestine, colon, liver, kidney and spleen) available from both species^48^. CHIA (CHIA1) mRNA was highly expressed in the stomach tissues of common marmoset (Fig. 6b, upper panel; Supplementary Table S2). Similarly, crab-eating monkey also expressed CHIA1 at high levels in this tissue (Fig. 6b, lower panel; Supplementary Table S2), in accordance with recent reports^47,48^.

In contrast, CHIA2 mRNAs were transcribed at very low levels in all tested common marmoset tissues, which were hundreds to thousand times lower than that of GAPDH (Fig. 6b, upper panel; Supplementary Table S2). Marmoset CHIA2 was near background level in the stomach tissues (60,000 and 20 times lower than that of CHIA (CHIA1) and CHIT1). Crab-eating monkey CHIA2 mRNA was expressed at low, but detectable levels in the tissues (Fig. 6b, lower panel; Supplementary Table S2), consistently with the previous report^47^. Thus, marmoset CHIA2 mRNA was lower than that of crab-eating monkey CHIA2 in this analysis (Fig. 6b, lower panel). Our results indicate that CHIA (CHIA1) gene is highly expressed in this organism with the primary location in the stomach tissues. CHIA2 may have other, non-digestive functions and could be expressed in tissues not tested in this study, during other life stages or under other circumstances.

## Discussion

In this study, we analyzed expression levels and enzymatic functions of CHIA in common marmoset. Our results show that the CHIA mRNA is highly expressed in the stomach and that the CHIA protein is resistant to proteases while degrading polymeric chitin substrates and mealworm shells into (GlcNAc)_2_. The activity was observed under conditions mimicking gastrointestinal environment, which is consistent to previous findings of omnivorous livestock and domestic animals^25–28,49^. In addition, we investigated whether both CHIA genes present in the marmoset genome are transcribed and showed that one of these genes is highly expressed in the stomach tissues. Thus, common marmoset is a good animal model for studying the nutritional values and physiological effects of insects and chitin as well as molecular evolution of the CHIA gene.

Common marmoset CHIA protein is most active at pH 2.0 and the activity decreases with increasing pH and lingered to up to pH 7.0, similarly chicken Chia^26^. In our previous report, we have shown that Chia expression and chitinolytic activity levels are much higher in omnivorous animals when compared to carnivorous and herbivorous animals^28^. Common marmoset is also omnivorous with CHIA highly expressed in the stomach while efficiently degrading chitin substrates under the GIT condition. These results indicate that the correlation between feeding behaviors and CHIA gene expression levels and enzymatic activity also apply to the insectivorous nonhuman primates. Besides our previous studies, two recent reports also suggested that CHIA expression in primates, including common marmoset, is related to feeding behavior and that CHIA genes may have been subjected to selection based on the diet^29,30^. The expression and activity of CHIA in the marmoset stomach described in this study is consistent with previously published data.

It has been shown that digestive enzymes have been subjected to natural selection due to adaptation to dietary environments of primates at molecular levels^50^. A recent study reported on evolution of CHIA being related to insectivory in nonhuman primates and that multiple CHIA genes are found in the nonhuman primate genomes^29^. For instance, black-and-white colobus species (herbivorous monkeys) do not have protein-coding CHIA gene, whereas *Tarsius syrichta*, an insectivorous primate, has five protein-cording CHIA genes in the genome. Other recent study suggested that there is no evidence of copy number variation in the CHIA genes in Old World and New World monkeys including common marmoset^51^. Our present results, together with previous studies^29,47^ indicate that common marmoset and crab-eating monkey highly express one CHIA gene (CHIA1) in the stomach tissues. These data suggest that both of these primates may still be evolving where one of the genes is dedicated to chitin digestion while the other one may be losing at least its digestive function.

In the normal marmoset stomach tissue, the CHIA expression level exceeded the expression of GAPDH, a well-known housekeeping gene. Wild common marmoset feeds on insects^29,34^, however, captive marmosets usually do not have chitin-containing in their diet (see the Methods). One may think that over-expressed CHIA can lead adverse effect on the marmoset stomach or body. However, chitin is not components of GIT or other tissues in mammals. Recently, mouse Chia was shown to be a constitutively produced enzyme essential for chitin degradation in the airways to maintain lung functions^21,22^. In addition, mouse Chia plays role in the protective immune response to gastrointestinal nematodes in the host GIT^23^. Thus, CHIA over-expressed in marmoset’s stomach do not have adverse effects to the body, rather it may maintain the level to support the healthy condition, in addition to chitin digestion.

Due to the effect on lipid metabolism and immunostimulation, chitin and chitosan (deacetylated version of chitin), are well accepted by animals and humans^6,52,53^. In addition, chitin and chitosan oligosaccharides (*N*-acetyl-chitooligosaccharides), prepared either chemically or enzymatically, have various biological activities such as anti-inflammatory properties^1,52–54^. According to our present and previous studies^25–28^, livestock and domestic animals can digest chitin, which has long been thought to be indigestible diets^24^. Nevertheless, there is a lack of research on the effects on growth and health performances when administrating chitin/chitosan as well as insects as diets. Further research is needed for introducing chitin/chitosan diets not only for animal feed resources but for human as supplements to enhance their health. Here we propose that a small nonhuman primate, common marmoset, could represent a suitable animal model for studying nutritional values of insects- and other chitin/chitosan-containing diets due to the simple handling and genetic similarities to human.

## Methods

### Common marmoset tissues

The marmoset colony was housed as described previously^36^. The marmosets were fed by balanced diet pellets (CMS-1M; CLEA Japan, Inc., Kawasaki, Japan), including L (+)-ascorbic acid (Nacalai Tesque, Tokyo, Japan), vitamins A, D3, and E (Duphasol AE3D; Kyoritsu Seiyaku Co., Ltd., Tokyo, Japan), and honey (Nihonhatimitsu Co., Ltd., Gifu, Japan). In addition, the marmosets were received supplemental foods consisting of sponge cakes and biscuits.

The study was conducted in accordance with the guidelines of Central Institute for Experimental Animals (CIEA) that comply with the Guidelines for Proper Conduct of Animal Experiments published by the Science Council of Japan. All animal experiments were approved by the Institutional Animal Care and Use Committee (CIEA ref. nos 12025 and 13071). Animal care was conducted in accordance with the Guide for the Care and Use of Laboratory Animals (Institute for Laboratory Animal Resources, 2011).

Ten marmoset tissues were dissected from a seven-year-old female marmoset (265 g, Animal I4704F). We also used other five stomach tissues (ten-year-old male, 282 g, I4170M; eight-year-old male, 274 g, I5057M; ten-year-old male, 239 g, I4370M; ten-year-old male, 242 g, R100817M; ten-year-old male, 268 g, I4347M). The sample collection was performed after euthanasia by exsanguination under ketamine (50 mg/kg) and xylazine (1.5 mg/kg) and isoflurane deep anesthesia. The tissues were frozen by liquid nitrogen and stored at −80 °C.

### RNA isolation and cDNA preparation

RNA was isolated from the marmoset ten tissues (brain, salivary, lung, heart, stomach, intestine, colon, liver, kidney and spleen) using TRIzol Reagent (Thermo Fisher Scientific, Waltham, MA, USA) according to the manufacturer’s instructions. cDNAs were synthesized from total RNA essentially as described previously^25–27,39^. Total RNA samples were treated with RQ1 RNase-Free DNase (Promega, Madison, WI, USA) according to the manufacturer’s protocol. Each of the total RNA samples (3 µg) was subjected to reverse transcription with random hexamers as primers. The reaction mixture (15 µl) contained enzyme buffer [50 mM Tris-HCl (pH 8.3), 75 mM KCl, and 3 mM MgCl_2_], 100 ng of random hexamers, 10 mM dithiothreitol, and 0.5 mM deoxynucleotide triphosphates (dNTPs). After heating the solution to 60 °C for 5 min and incubating the mixture at 37 °C for 5 min, 200 U of recombinant murine leukemia virus reverse transcriptase (Thermo Fisher Scientific) was added, and the mixture was incubated at 37 °C for 45 min. The reverse transcription was terminated by heating the samples to 95 °C for 5 min.

### Primers for qPCR

We designed primers for qPCR using PrimerQuest Input (Integrated DNA Technologies, Coralville, IA, USA) and evaluated their suitability based on a single product generation, which are reflected by a single melting temperature as described previously^25–27,39,40^. The nucleotide sequences of the primers selected for qPCR are shown in Supplementary Table S3.

### Standard DNA for qPCR

We designed a five genes standard DNA coding for CHIA, CHIT1, GAPDH, pepsinogen A and H^+^/K^+^-ATPase. It was commercially synthesized and inserted into pTAKN-2 vector (Eurofins Genomics, Tokyo, Japan). We prepared the standard DNA (399 bases; see Supplementary Fig. S1) amplified by PCR from the plasmid DNA using the forward primer 5′-GTGGCCTGTACCCTGACC-3′ and the reverse primer 5′-GTCACAATGGAGGCAAAGTTATC-3′.

### qPCR

PCR reactions were performed in a final volume of 13 µl containing 2 x SYBR Green Master Mix (Brilliant II SYBR Green QPCR Master Mix, Agilent, Santa Clara, CA, USA), 2.7 ng of marmoset cDNA or appropriate dilutions of the external standards and 2.3 pmol of the primers listed in Supplementary Table S3^25–27,39^. Standard real-time PCR conditions for the Mx3005P (Agilent) were used: an initial denaturation and polymerase activation step for 10 min at 95 °C, followed by 40 cycles of denaturation at 95 °C for 30 sec, annealing at 55 °C for 30 sec and polymerization at 72 °C for 10 sec. We performed each reaction in triplicate.

### Preparation of common marmoset stomach extract

Stomach tissue isolated from a 7-year-old female marmoset was homogenized in 10 volumes of ice-cold TS buffer [20 mM Tris-HCl (pH7.6), 150 mM NaCl] using a Teflon/glass homogenizer. The homogenates were centrifuged at 15,000 g for 10 min at 4 °C and the supernatants were kept. The soluble fraction was used as the stomach extract. We pre-incubated the extract at 37 °C for 0, 5, 10, 30 or 60 min at pH 7.6 or pH 2.0. After incubation, followed by addition of inhibitor (Complete Mini, Roche, Basel, Switzerland), the solutions were neutralized by addition of 1 M Tris-HCl (pH 7.6). Then, equal amount (5 µg) of a 1:1 mixture of the trypsin (Sigma-Aldrich, St. Louis, MO, USA) and chymotrypsin (Sigma-Aldrich) was added to the reaction mixture. The reaction mixtures were incubated at 37 °C for 1 hour at pH 7.6.

### Antibody preparation

Rabbit polyclonal antibodies to pig Chia was generated by Eurofins Genomics. Cys-peptides were conjugated through the added N-terminal cysteine to keyhole limpet hemocyanin (KLH). Sera from immunized rabbits were affinity-purified using the antigen with Cys (pig Chia, CMREAFEQEAKQTK) coupled to Sulfolink (Thermo Fisher Scientific).

### SDS-PAGE, CBB staining and Western blot

We analyzed the protein fractions using standard SDS-PAGE, followed by Coomassie Brilliant Blue R-250 (Sigma-Aldrich) or Western blot. Separated proteins were transferred to a polyvinylidene fluoride (PVDF) membrane (Immobilon-P, Merck Millipore, Tokyo, Japan), which was probed using polyclonal anti-pig pepsin antibody (GeneTex, Irvine, CA, USA) or polyclonal anti-pig Chia, followed by incubation with horseradish peroxidase conjugated secondary antibodies of AffiniPure Donkey Anti-Goat IgG-HRP (Jackson ImmunoResearch laboratories) or AffiniPure F (ab’)_2_ Fragment Donkey Anti-Rabbit IgG (H + L) (Jackson ImmunoResearch Laboratories, Inc., West Grove, PA, USA). We analyzed and quantified the immunoblots using Luminescent Image Analyzer (ImageQuant LAS 4000, GE Healthcare, Piscataway, NJ, USA).

### Chitinase assays

We determined chitinolytic activity using 4-nitrophenyl *N*,*N*′-diacetyl-β-D-chitobioside (4-NP-chitobioside, Sigma-Aldrich), at a concentration of 200 µM. Each reaction was performed in triplicate^55^. All enzymatic reactions were performed using total protein extract of marmoset stomach in Gly-HCl buffer (pH 1.0 to pH 3.0) or McIlvaine’s buffer (0.1 M citric acid and 0.2 M Na_2_HPO_4_; pH 2.0 to pH 8.0) in a volume of 50 µL. After incubation at 37 °C for 30 min, 20 µL of 1 M sodium carbonate solution was immediately added to the reaction mixture. The absorbance of the released 4-nitrophenolate ion was measured at 405 nm. A molar extinction coefficient for 4-nitrophenol of 17,700 M^−1^ cm^−1^ was used in the calculations.

One enzyme unit (U) was defined as 1 μmol of 4-nitrophenol released from 4-NP-chitobioside per min at 37 °C in Gly-HCl buffer (pH 2.0).

### Influence of pH and temperature on the chitinase activity of CHIA

To determine the optimal pH, we measured the chitinase activity by incubating the samples with 4-NP-chitobioside in 0.1 M Gly-HCl buffer (pH 1.0 to pH 3.0) or McIlvaine’s buffer (0.1 M citric acid and 0.2 M Na_2_HPO_4_; pH 2.0 to pH 8.0) at 37 °C for 30 min. To determine the optimal temperature, we assayed the chitinase activity between 30 °C and 65 °C in 0.1 M Gly-HCl buffer (pH 2.0).

To determine the pH stability, we incubated the samples for 1 hour on ice in 0.1 M Gly-HCl buffer (pH 1.0 to pH 3.0) and McIlvaine’s buffer (pH 2.0 to pH 8.0). After the pre-incubation, we measured the residual activity at pH 2.0 in 0.1 M Gly-HCl buffer, as described above.

For heat stability measurement, we incubated the samples in 0.1 M Gly-HCl buffer (pH 2.0) for 15 min between 30 °C and 65 °C. After cooling on ice, we measured the activity in 0.1 M Gly-HCl buffer (pH 2.0), as described above.

### Preparation of shells from mealworms

We purchased mealworm (*Tenebrio molitor*) larvae from local commercial supplier (Lumberjack Co., Ltd., Tokyo, Japan) and used the shells as chitin-protein polymer substrates as described previously^26,27^.

### Digestion of chitin substrates and mealworm larvae shell

The enzymatic reactions under conditions mimicking stomach or intestine environment using α, β and colloidal chitin substrates (at a final concentration of 1 mg/mL) were performed in a volume of 50 µL containing 4 µL of 200 mU/mL soluble protein from common marmoset stomach at pH 2.0 and 37 °C for 1 hour or at pH 7.6 with trypsin and chymotrypsin and 37 °C for 1 hour. We incubated the mealworm larvae shells with 10 µL of 200 mU/mL soluble protein fraction in an analogous way except for further incubation (total 20-hours) in intestine-like condition. Chitin fragments generated in the gastrointestinal conditions were analyzed by FACE method as originally described by Jackson^45^ and recently improved by us^46^. *N*-acetyl chitooligoaccharides (Seikagaku Corporation, Tokyo, Japan) were used as a standard.

### Quantification of CHIA (CHIA1) and CHIA2 mRNAs by qPCR

Crab-eating monkey Total RNA Panel was purchased from Funakoshi Co., Ltd. Total RNA was reverse-transcribed into cDNA essentially as reported recently^48^.

For evaluating expression of CHIA (CHIA1) and CHIA2 mRNAs in both marmoset and crab-eating monkey tissues, we set up a quantification system to compare the mRNA levels of multiple genes between a number of common marmoset and crab-eating monkey tissues using a common marmoset and crab-eating monkey hybrid standard DNA (Supplementary Fig. S8). We listed the primers’ sequences in Supplementary Table S4. QPCR was carried out as described above.

### Statistical analysis

qPCR, protein concentration, enzymatic activity and immunoblotting data were compared by Student’s t-test.

## Electronic supplementary material

Supplementary Information

## Data Availability

The datasets generated and/or analyzed during the current study are available from the corresponding author on reasonable request.
