# Supplementary material for: High expression of acidic chitinase and chitin digestibility in the stomach of common marmoset (Callithrix jacchus), an insectivorous nonhuman primate
Source: Sci Rep. 2019 Jan 17;9:159. doi: 10.1038/s41598-018-36477-y (PMC6336882; doi:10.1038/s41598-018-36477-y)
Supplement: Supplementary file 1 — Supplementary Information [file 41598_2018_36477_MOESM1_ESM.pdf]

Supplementary Information

High expression of acidic chitinase and chitin digestibility in the stomach of common marmoset (*Callithrix jacchus*), an insectivorous nonhuman primate

Eri Tabata[1,2], Akinori Kashimura[1], Maiko Uehara[1], Satoshi Wakita[1], Masayoshi Sakaguchi[1], Yasusato Sugahara[1], Terumi Yurimoto[3], Erika Sasaki[3], Vaclav Matoska[4], Peter O. Bauer[4, 5] & Fumitaka Oyama[1, *]

[1]Department of Chemistry and Life Science, Kogakuin University, Hachioji, Tokyo 192-0015, Japan, [2]Research Fellow of Japan Society for the Promotion of Science (DC1), Koujimachi, Chiyoda-ku, Tokyo 102-0083, Japan, [3]Central Institute for Experimental Animals, Tonomachi, Kawasaki, Kanagawa, 210-0821, Japan, [4]Laboratory of Molecular Diagnostics, Department of Clinical Biochemistry, Hematology and Immunology, Homolka Hospital, Roentgenova 37/2, Prague 150 00, Czech Republic, [5]Bioinova Ltd., Videnska 1083, Prague 142 20, Czech Republic

(a)

CHIA	CHIT1	GAPDH	Pep A	H^+/K^+-ATPase

(b)

GTGGCCTGTACCCTGACCCCACTGACAAGAATGCCTTCTACCACTGTTTG
AATGGAAAGACTTTCATCCAGCACTGCCAGACTGGCCTTGTCTTCGATGC
C**TCCTGCTCCTGCTGCAACTGGGTCAACTCAGCCATCAGGTT**TCTGCGCA
AATACGGC**TTTGACGGTCTTGACCTTGATGGAAATCCCATCACCATCTT**C
CAGGAGCGAGATCCCT**CCAAAATCAAGTGGGGCGTACCCTGTGCCAGCCA**
GTGCCTACATCCTGCAGGACGAGGGG**GGCTGCACCAGCGGCTTCCAAGAA**
GGCAGACATTGGAGTAGCCATGGGCATTGCTGGCTCAGATGCTGCCAAAA
ATGCAGCCGACATGATCCTGCTGGAT**GATAACTTTGCCTCCATTGTGAC**

Supplementary Figure S1. Standard DNA molecule used for qPCR in Fig. 1. (a) Schematic representation of the standard DNA molecule. (b) Nucleotide sequence of the standard DNA. The standard DNA, 399 bases long, contained cDNA fragments of CHIA, CHIT1, GAPDH, pepsinogen A (Pep A) and H^+/K^+-ATPase in a one-to-one ratio. Primers for qPCR in each target DNA region are shown in underline.

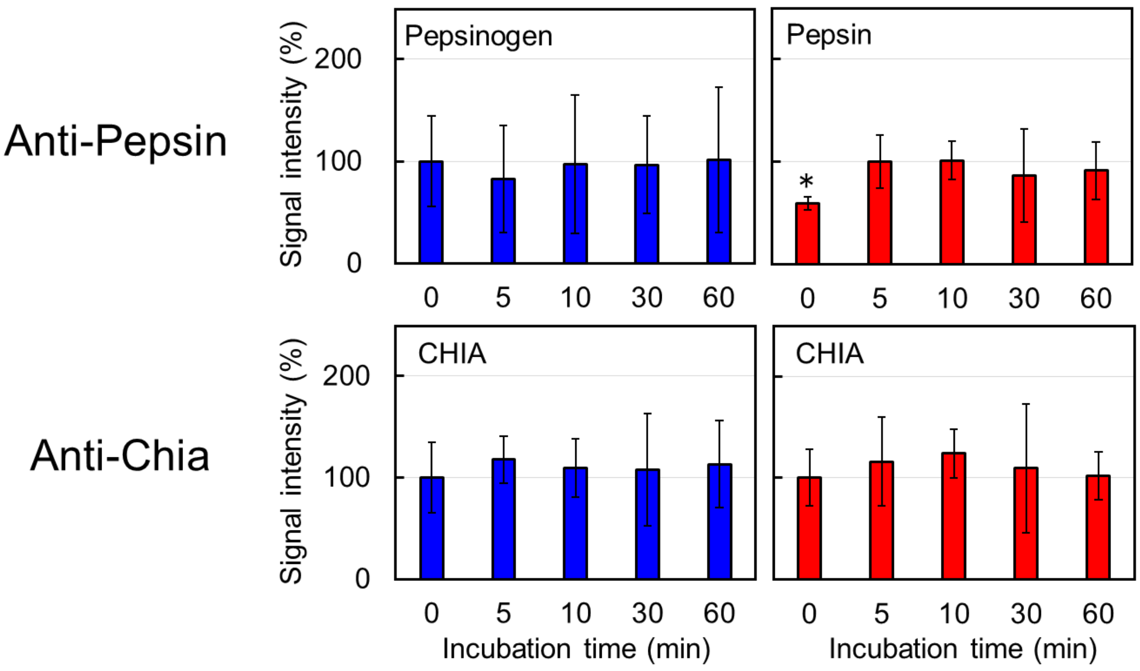

Supplementary Figure S2. Quantification of the protein in Fig. 2b. The signal intensities were quantified as described in the Methods. Soluble proteins obtained from common marmoset stomach were incubated at pH 7.6 (blue) or 2.0 (red) and Western blot analysis was performed as described in the Fig. 2b. The signal intensities of pepsinogen (pH 7.6) and CHIA (pH 7.6 and pH 2.0) at 0 min and of pepsin (pH 2.0) at 5 min of incubation were defined as 100 %. Values represent mean ± SD, experiments were conducted in triplicate. *p< 0.05. P-values were determined using Student’s t-test.

Pig Chia

YQLICYFTNWAQYRPGLGSFKPDDIDPCLCTHLVYAFAGMRDNEITTTEGDDVTFYQSF
NGLKNKNSQLKTLLAIGGWNFGTAPFTAMVSAAENRQTFITSVIKFLRQYGFDGLDFDW
EYPGSRGSPSQDKHLFTVLVQEMREAFEQEAKQTKQARLLVTAAVAAGVSNIQSGYEIP
QLSQYLDYIHVMTYDLHASWEGYAGENSPLYKYPTDTGSNAYLNVDYAMNYWKDNGAPA
EKLIVGFPAYGHTFLLSNPSNTDIGAPTSGAGPAGPYTKEAGFWAYYEICTFLKNGATQ
AWDAPQDVPYAYKGNEWVGYDNVKSFNIKAQWLKQNNFGGAMVWAIDLDDFTGTFCNQG
KFPLINTLKDALGLNSTSCTASAQPSEPSSGTGSGSTTGSGSGSSSSGSSSGGSGYCAG
KADGLYPVANNRNAFWHCQNGITYEQYCQTGLVFDTSCQCCNWA

Marmoset CHIA

YQLTCYFTNWAQYRPGLGRFKPDDIDPCLCTHLIYAFAGMRNNEITTIEWDDLTLYQAF
NGLKNKNSELKTLLAIGGWNFGTAPFTAMVSTPENRQTFITSVIKFLRQYEFDGLDFDW
EYPGSRGSPPQDKHLFTVLVQEMREAFEQEAKQINKPRLMVTAAVAAGISNIQSGYEIP
QLSQYLDYIHVMTYDLHGSWEGYTGENSPLYKYPTDTGSNAYLNVDYAMNYWKDNGAPA
EKLIVGFPAYGHTFLLSNPSNHGIGASTTGPGPAGPYTRQSGFWAYYEICTFLKDGATE
VWEAPEDVPYAYKGNEWLGYDNTKSFKIKADWLKTNNFGGAMVWAIDLDDFTGTFCNQG
KFPLITTLKDALGLQSASCKAPAQPIAPITEAPSTGGVSHSGSSGSSSGSSPSGSGFCA
NRASGLYPDPTDKNAFYHCLNGKTFIQHCQTGLVFDASCSCCNW

Supplementary Figure S3. Locations and sequences of the antigen used for the anti-pig Chia antibody in the mature pig Chia and marmoset CHIA proteins. The antigen sequences are shown in red. The same sequence (shown in red and underlined) is also present in the marmoset CHIA.

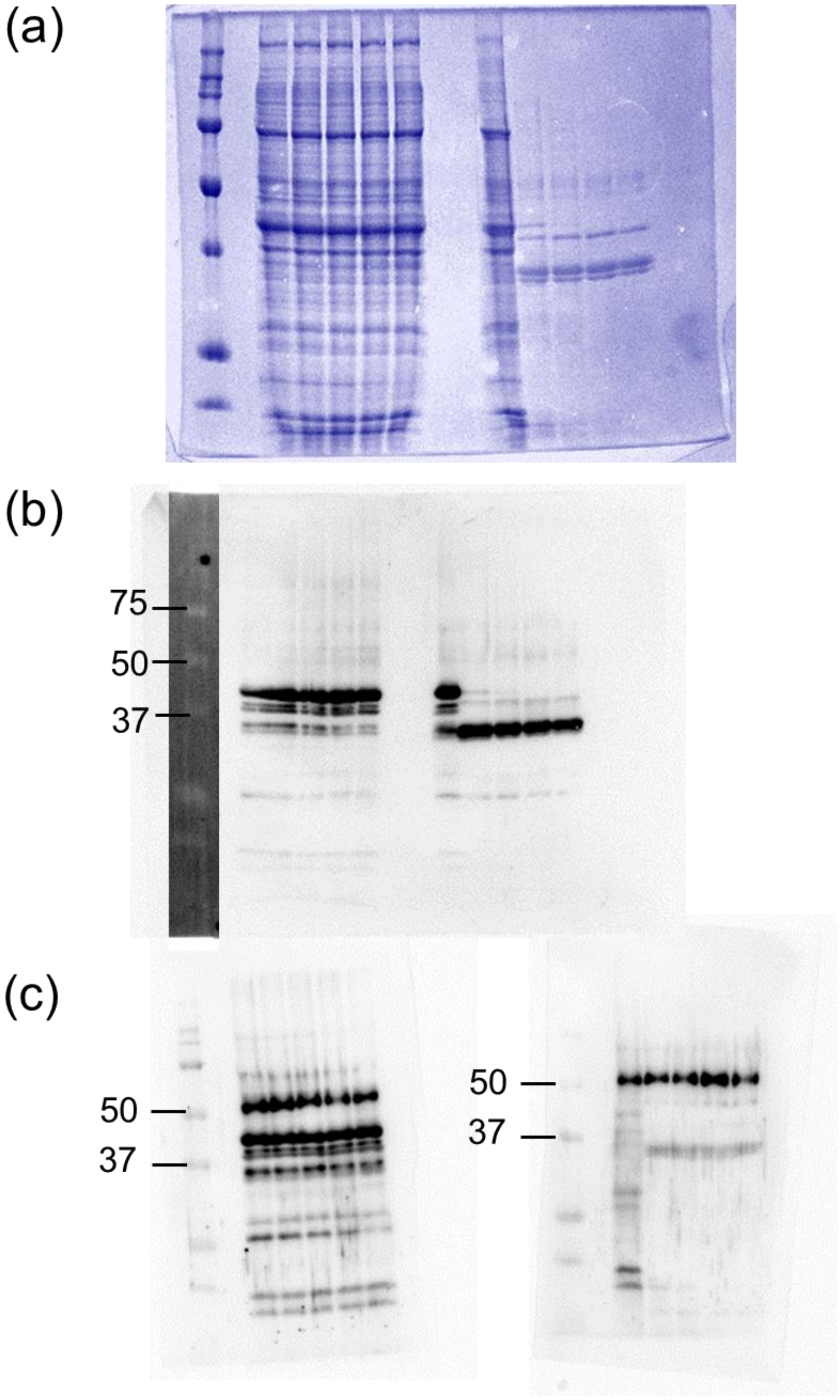

Supplementary Figure S4. Full-length gel and blots shown in Fig. 2. (a) Full-length gel images of SDS-PAGE and CBB staining. (b) Western blotting using anti-pepsin antibody. (c) Western blotting using anti-CHIA antibody.

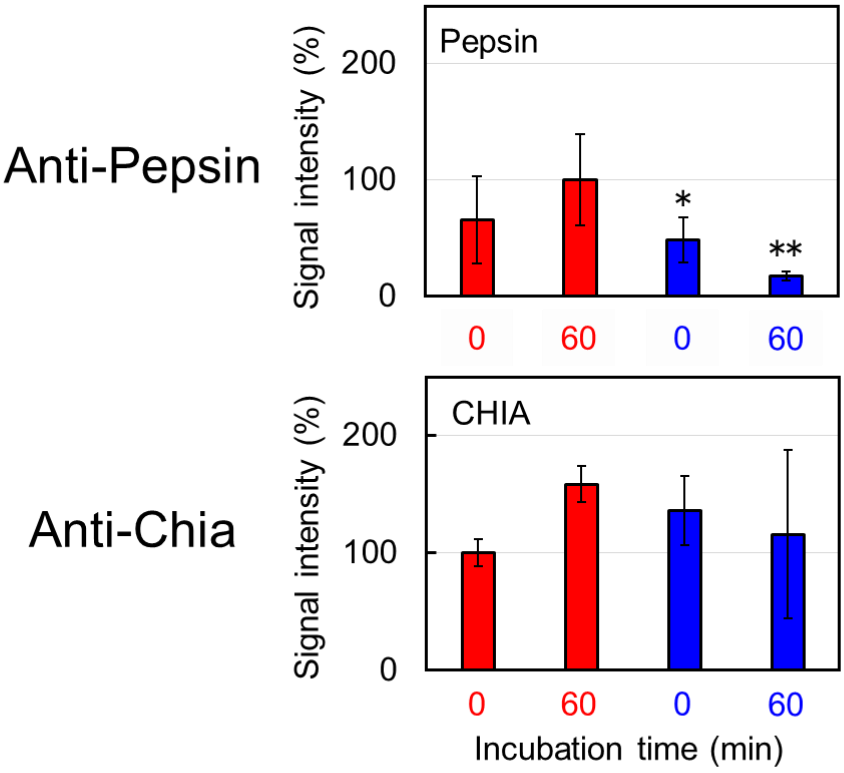

Supplementary Figure S5. Quantification of the protein in Fig. 4. The signal intensities were quantified as described in the Methods. Soluble proteins obtained from common marmoset stomach were incubated at pH 2.0 (red) for 60 min, neutralized and further incubated at pH 7.6 (blue) with trypsin/chymotrypsin for 60 min followed by Western blot analysis as described in the Fig. 4. The signal intensities of pepsin (pH 2.0) and CHIA (pH 2.0) at 0 min were defined as 100 %. Values represent mean ± SD, experiments were conducted in triplicate. **p*< 0.05, ***p*< 0.01. P-values were determined using Student's t-test.

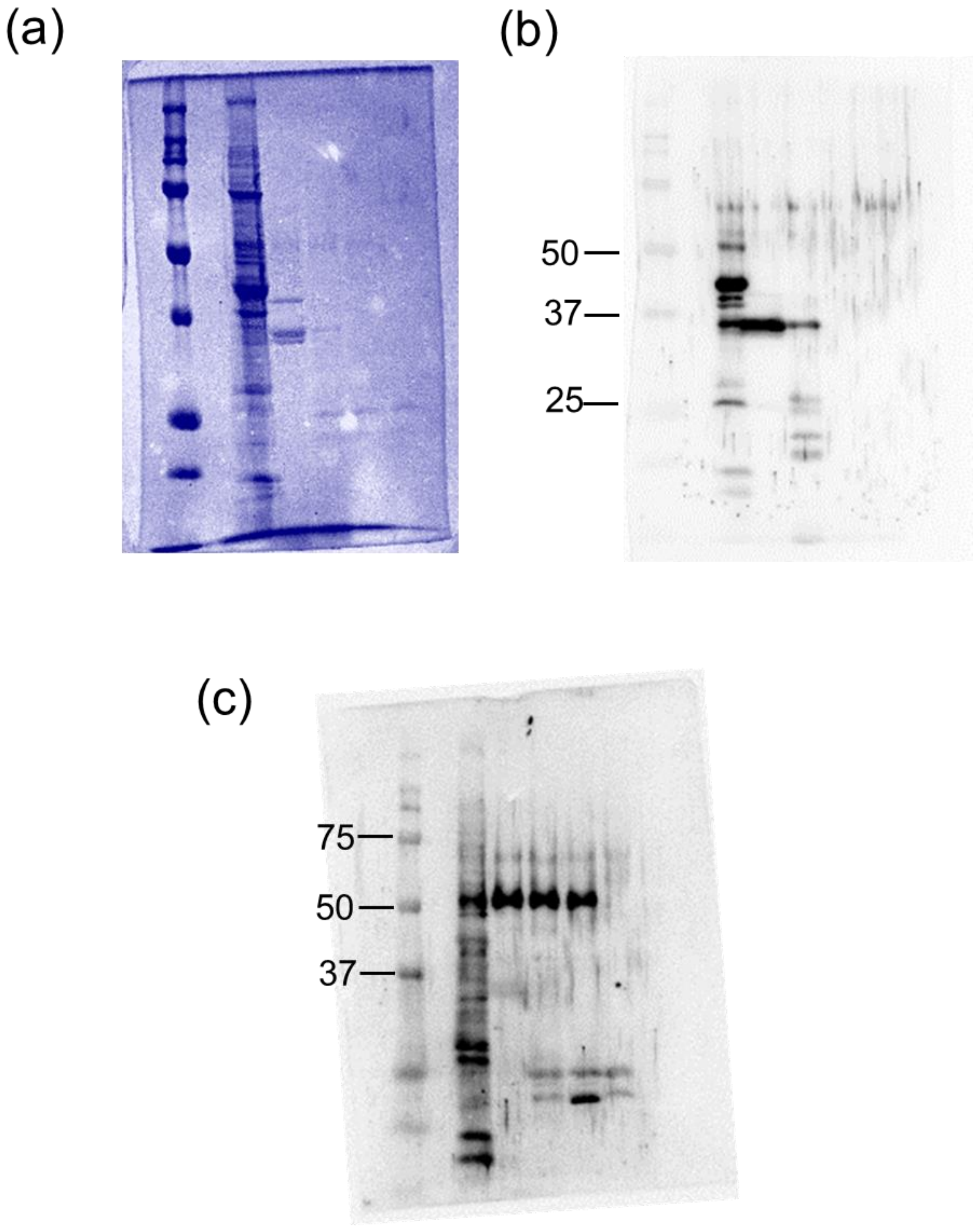

Supplementary Figure S6. Full-length gel and blots shown in Fig. 4. (a) Full-length gel images of SDS-PAGE and CBB staining. (b) Western blotting using anti-pepsin antibody. (c) Western blotting using anti-CHIA antibody.

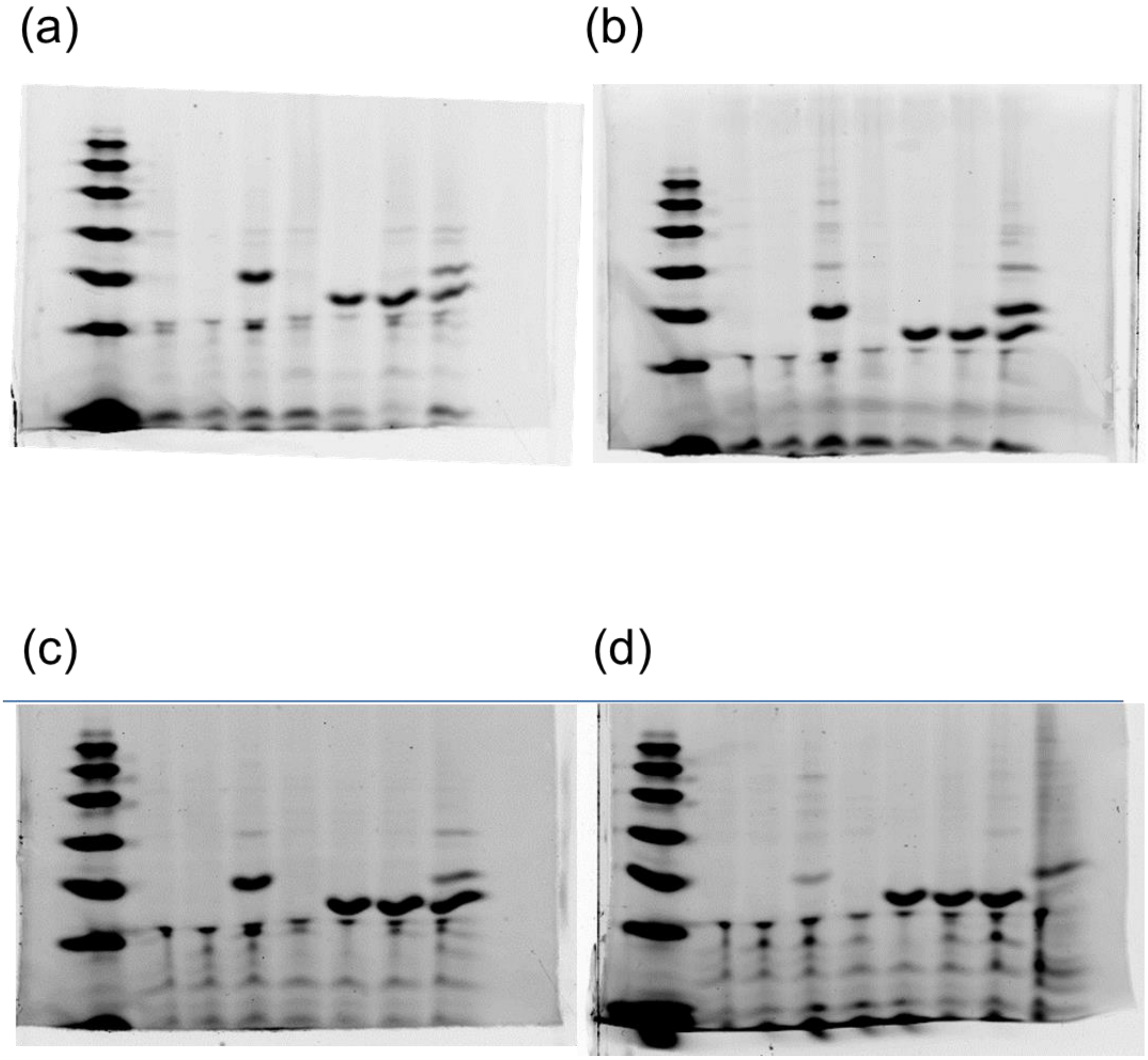

Supplementary Figure S7. Full-length gel and blots shown in Fig. 5. (a-d) Full-length gel images of the FACE methods in Fig. 5a-d.

(a)

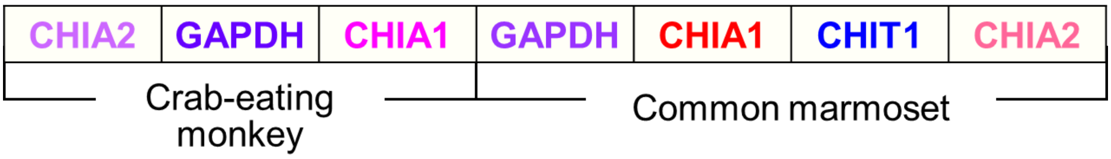

(b)

GGGCTTACTATGAGATCCTGAAAAATGGAGCCACTCAGGAATGGGATG**AC**
CCTAAGAAAGTGCCTTATGCCTTTGGTATCGTGGAAGGACTCATGACCAC
AGTCCACGCCATCACTGCCACCCAGAAGACTGTGGATGGCCCCTCCGGGA
AACTGTGGCGTGATGGCCGCGGAGCTCTCCAGA**ACATCATCCCTGCCTCT**
ACTGAATTCCTGCGCCAGTATGAGTTTGATGGGCTGGACTTCGACTGGGA
GTACCCCGGCTCTCGTGGGAGCCCTTCTCAGGACAA**GCATCTCTTCACTG**
TCCTGGTGCAATGGAAATCCCATCACCATCTTCCAGGAGCGAGATCCCT**C**
CAAAATCAAGTGGGGCGGTGGCCTGTACCCTGACCCCACTGACAAGAATG
CCTTCTACCACTGTTTGAATGGAAAGACTTTCATCCAGCACTGCCAGACT
GGCCTTGTCTTCGATGCC**TCCTGCTCCTGCTGCAACTGGGTCAACTCAGC**
CATCAGGTTTCTGCGCAAATACGGC**TTTGACGGTCTTGACCTTGCAATAC**
CCGACGGACAACCTCAATGTGGATTATGTCATGAACTACTGGAAGGACAA
CGGGGCCCCAGCTAAGAAGCTCATTGTTGGATTCCCTACCTATGGACGCA
CCTTC**ACCCTGAGCAACCCTTCC**

Supplementary Figure S8. Standard DNA molecule used for qPCR in Fig. 6b. (a) Schematic representation of the standard DNA molecule. (b) Nucleotide sequence of the standard DNA. The standard DNA, 673 bases long, contained cDNA fragments of crab-eating monkey [CHIA2, GAPDH and CHIA (CHIA1)] and common marmoset (GAPDH, CHIA1, CHIT1 and CHIA2) in a one-to-one ratio. Primers for qPCR in each target DNA region are shown in underline.

Supplementary Table S1. qPCR analysis data in Fig. 1.
(a)
GAPDH

	Molecules				
Tissue	Exp. 1	Exp. 2	Exp. 3	Average	SD
Brain	27,240	27,050	26,940	27,077	152
Salivary	1,817	1,938	1,950	1,902	74
Lung	1,255	1,450	1,355	1,353	98
Heart	6,055	6,003	5,826	5,961	120
Stomach	1,984	2,129	2,048	2,054	73
Intestine	2,520	2,471	2,409	2,467	56
Colon	4,010	4,559	4,478	4,349	296
Liver	4,634	4,799	4,432	4,622	184
Kidney	4,316	4,823	4,462	4,534	261
Spleen	991	1,099	1,188	1,093	99

CHIT1

	Molecules				
Tissue	Exp. 1	Exp. 2	Exp. 3	Average	SD
Brain	52	54	74	60	12
Salivary	21	59	16	32	24
Lung	386	371	366	374	10
Heart	15	8	4	9	5
Stomach	13	10	13	12	2
Intestine	9	15	9	11	4
Colon	8	2	4	5	3
Liver	374	331	342	349	22
Kidney	39	13	6	19	17
Spleen	162	128	134	141	18

CHIA (CHIA1)

	Molecules				
Tissue	Exp. 1	Exp. 2	Exp. 3	Average	SD
Brain	94	11	6	37	49
Salivary	43	9	5	19	21
Lung	11	4	7	7	3
Heart	13	6	15	11	5
Stomach	54,030	49,750	46,290	50,023	3,877
Intestine	3	1	2	2	1
Colon	50	1	1	17	28
Liver	42	4	18	21	19
Kidney	35	23	21	27	8
Spleen	8	5	1	5	3

(b)

	Molecules							
Gene	I4170M	I5057M	I4370M	R100817M	I4347M	I4704F	Average	SD
CHIT1	22	3	22	11	9	15	14	7
CHIA (CHIA1)	48,770	123,400	123,000	57,740	38,430	65,510	76,142	37,556
Pepsinogen A	1,984,000	4,764,000	6,103,000	2,877,000	3,234,000	1,319,000	3,380,167	1,777,698
H^+/K^+-ATPase	28,530	42,160	50,830	25,030	18,100	15,040	29,948	13,957
GAPDH	6,125	5,921	17,050	8,429	7,216	2,721	7,910	4,865

Supplementary Table S2. qPCR analysis data in Fig. 6 (b_1)

Marmoset CHIA (CHIA1)

	Molecules				
Tissue	Exp. 1	Exp. 2	Exp. 3	Average	SD
Brain	4	8	6	6	2
Lung	4	4	7	5	2
Stomach	19,860	22,930	24,900	22,563	2,540
Intestine	4	5	6	5	1
Colon	7	6	5	6	1
Liver	3	5	5	4	1
Kidney	27	8	6	14	12
Spleen	5	5	9	6	2

Marmoset CHIA2

	Molecules				
Tissue	Exp. 1	Exp. 2	Exp. 3	Average	SD
Brain	3	7	3	4	3
Lung	4	4	2	3	1
Stomach	3	4	3	3	0.5
Intestine	4	3	2	3	1
Colon	2	4	1	2	1
Liver	3	3	0	2	2
Kidney	2	4	1	2	1
Spleen	3	2	2	2	0.3

Marmoset GAPDH

	Molecules				
Tissue	Exp. 1	Exp. 2	Exp. 3	Average	SD
Brain	22,020	24,020	23,210	23,083	1,006
Lung	1,160	1,069	972	1,067	94
Stomach	2,556	2,847	2,857	2,753	171
Intestine	1,837	2,018	2,261	2,039	213
Colon	3,175	3,264	3,293	3,244	61
Liver	3,829	3,671	3,711	3,737	82
Kidney	3,735	4,259	4,061	4,018	265
Spleen	649	610	569	609	40

Marmoset CHIT1

	Molecules				
Tissue	Exp. 1	Exp. 2	Exp. 3	Average	SD
Brain	107	132	98	112	18
Lung	8	7	5	7	2
Stomach	5	6	4	5	1
Intestine	3	3	2	3	1

Colon	124	111	104	113	11
Liver	105	95	101	100	5
Kidney	5	3	5	4	1
Spleen	11	13	26	17	8

Marmoset Stomachs

	Molecules							
Gene	I4170M	I5057M	I4370M	R100817M	I4347M	I4704F	Average	SD
CHIA (CHIA1)	37,600	79,170	82,840	36,390	27,030	48,060	66,537	25127
CHIA2	1	1	0	N.D.	2	N.D.	1	1
CHIT1	31	9	19	15	5	12	20	11
GAPDH	6,741	5,939	17,860	9,193	8,313	3,264	10,180	6663

(b_2)
Crab-eating monkey CHIA1

	Molecules				
Tissue	Exp. 1	Exp. 2	Exp. 3	Average	SD
Brain	10	8	21	13	7
Lung	12	15	9	12	3
Stomach	141,600	127,600	133,800	134,333	7,015
Intestine	11	7	16	11	5
Colon	15	7	13	12	4
Liver	23	17	24	21	4
Kidney	14	11	13	13	2
Spleen	11	10	7	9	2

Crab-eating monkey CHIA2

	Molecules				
Tissue	Exp. 1	Exp. 2	Exp. 3	Average	SD
Brain	63	87	63	71	14
Lung	71	66	64	67	4
Stomach	74	80	73	76	4
Intestine	63	59	70	64	6
Colon	63	50	71	61	11
Liver	68	75	71	71	4
Kidney	64	64	63	64	1
Spleen	62	75	46	61	14

Crab-eating monkey GAPDH

	Molecules				
Tissue	Exp. 1	Exp. 2	Exp. 3	Average	SD
Brain	15,520	14,940	13,700	14,720	930
Lung	4,102	3,088	2,902	3,364	646
Stomach	3,658	3,487	3,364	3,503	148
Intestine	9,140	8,654	9,358	9,051	360
Colon	3,329	3,481	3,457	3,422	82
Liver	6,145	5,474	5,916	5,845	341
Kidney	16,950	15,250	15,100	15,767	1,028
Spleen	4,909	5,193	4,530	4,877	333

Supplementary Table S3. List of qPCR primers.

Gene	Forward primer	Reverse primer
CHIA	GTGGCCTGTACCCTGACC	CCAGTTGCAGCAGGAGCAGGA
CHIT1	GTCAACTCAGCCATCAGGTT	CAAGGTCAAGACCGTCAAA
GAPDH	ATGGAAATCCCATCACCATCTT	CGCCCCACTTGATTTTGG
Pepsinogen A	TACCCTGTGCCAGCCAGTG	GGAAGCCGCTGGTGCAGCC
H^+/K^+-ATPase	AAGAAGGCAGACATTGGAGTAG	GTCACAATGGAGGCAAAGTTATC

Supplementary Table S4. List of qPCR primers for common marmoset CHIA (CHIA1), CHIA2, CHIT1 and GAPDH and crab-eating monkey CHIA1, CHIA2 and GAPDH.

Gene	Forward primer	Reverse primer
Marmoset_CHIA (CHIA1)	GTGGCCTGTACCCTGACC	CCAGTTGCAGCAGGAGCAGGA
Marmoset_CHIA2	CAATACCCGACGGACAAC	GGAAGGGTTGCTCAGGGT
Marmoset_CHIT1	GTCAACTCAGCCATCAGGTT	CAAGGTCAAGACCGTCAAA
Marmoset_GAPDH	ATGGAAATCCCATCACCATCTT	CGCCCCACTTGATTTTGG
Crab-eating monkey_CHIA1	GAATTCCTGCGCCAGTATGA	TGCACCAGGACAGTGAAGAGATG
Crab-eating monkey_CHIA2	GGGCTTACTATGAGATCCTGAAA	GCATAAGGCACTTTCTTAGGGT
Crab-eating monkey_GAPDH	CTTTGGTATCGTGGAAGGACTC	AGTAGAGGCAGGGATGATGT
